# The Stryker Regenkit PRP Kit Does Not Concentrate Canine Platelets

**DOI:** 10.3389/fvets.2020.555909

**Published:** 2020-10-22

**Authors:** Barry Cherno, Leilani Alvarez, Kenneth E. Lamb, Sherman Canapp

**Affiliations:** ^1^Integrative and Rehabilitative Medicine Department, Animal Medical Center, New York, NY, United States; ^2^Lamb Statistical Consulting LLC, West Saint Paul, MN, United States; ^3^Veterinary Orthopedic and Sports Medicine, Annapolis Junction, MD, United States

**Keywords:** platelet rich plasma (PRP), Stryker, Regenkit, autologous, PRP

## Abstract

Platelet Rich Plasma (PRP) works to recruit growth factors, anti-inflammatory mediators, and blood supply to an area that may not heal well under normal conditions (e.g., joints, tendons, ligaments). Previous research has demonstrated that various PRP systems create vastly different final products. The objective of this study was to evaluate the cellular composition of the final plasma product using the Stryker RegenKit system in dogs. A peripheral blood sample was obtained from ten healthy adult dogs and compared to the final plasma product. A standard CBC was performed prior to testing and an additional sample was processed according the manufacturing guidelines for obtaining PRP (10 ml total blood). Comparisons of mean platelet count, erythrocyte count, and leukocyte count were made between the peripheral blood sample and the final plasma product. Results showed that there was no significant difference in platelet count between the peripheral blood CBC and final PRP CBC (*p* = 0.349). There were significantly fewer erythrocytes and leukocytes in the final plasma product when compared with peripheral blood (*p* = < 0.0001, *p* = 0.0318, respectively). These results indicate that the Stryker Regenkit PRP Kit decreases leukocyte and erythrocyte concentrations but does not consistently change platelet concentrations in its final plasma product. This study, in combination with results from similar studies, may allow clinicians to better choose the appropriate PRP system to treat various musculoskeletal conditions in dogs.

## Introduction

There has been a growing interest by both pet owners and clinicians in regenerative medicine, specifically platelet rich plasma. Platelet Rich Plasma (PRP) is becoming increasingly common in canine medicine for the treatment of soft tissue injury, wound healing, bone healing, tendon and ligament injury, and for patients experiencing clinical symptoms of osteoarthritis (1–9). The benefit of PRP administration is attributed to the high concentration of growth factors contained within the alpha granules released when platelets are activated. These alpha granules release a variety of growth factors, including but not limited to, platelet-derived growth factor, insulin-like growth factor, transforming growth factor-β1 and β2, vascular endothelial growth factor, basic fibroblastic growth factor, and epidermal growth factor (10–12).

It is generally accepted that there are four classifications of Platelet Rich Plasma (13). These can be described as: Pure PRP or leukocyte-poor platelet rich plasma (P-PRP), Leukocyte- and Platelet-rich Plasma (L-PRP), Pure Platelet-Rich Fibrin or Leukocyte-Poor Platelet-Rich Fibrin (P-PRF), and Leukocyte- and Platelet-Rich Fibrin (L-PRF). Pure PRP, which was originally used for transfusions, typically contains 0.5 × 10^11^ platelets per unit (13). These can be injected (intra-articular) or used topically (as a gel for wounds). These concentrates are most commonly produced using continuous flow plasmapheresis and are devoid of leukocytes and have a low-density fibrin network. Leukocyte- and Platelet-rich Plasma (L-PRP) also have a low-density fibrin network; however, unlike Pure PRP, this concentration contains leukocytes. This type of PRP can be either injected or used topically. Pure Platelet-Rich Fibrin (P-PRF) has a high-density fibrin network and no leukocytes. These preparations are more of a solid consistency and therefore are used topically as a gel and cannot be injected. Finally, Leukocyte- and Platelet-Rich Fibrin (L-PRF) is another high-density fibrin network. Unlike P-PRF, these concentrates do contain leukocytes. Similarly, to P-PRF, due to the high-density fibrin network, these preparations are more solid and used topically and are not capable of being injected (14). Some will further categorize PRP into leukocyte-rich PRP (LR-PRP) vs. leukocyte-poor PRP (LP-PRP). LR-PRP is defined as having a neutrophil concentration above baseline while LP-PRP has a neutrophil concentration below baseline (15). Mishra et al. (16) went a step further in classifying PRP products, not only by the presence of leukocytes, but by separating those that contain > 5 times the platelet concentration compared to whole blood (Type A) from those than contain <5 times the platelet concentration compared to whole blood (Type B).

Many systems exist to process a PRP product in human and equine medicine fields as well as multiple canine specific systems. These systems have been shown to produce products differing significantly in platelet, red blood cell (RBC), and white blood cell (WBC) concentrations (12, 17–19). Each commercially available PRP system comes with its own set of instructions regarding preparation. One of the most important aspects of producing a PRP product is centrifugation. This process is what separates the different cell types (erythrocytes, leukocytes, and platelets) by gravitational force (G) into layers based on density so that they can be preferentially selected for what the manufacturer suspects to be the most ideal PRP concentrate. The G is determined by the revolutions per minute (RPM) and radius of the rotor. The time and RPM each tube of blood and anticoagulant is subject to is different between the systems. Some systems use a single “soft spin” (slower rotations, lower G) or single “hard spin” (faster rotations, higher G), while others undergo a combination or double spin (often a soft spin followed by removal of the erythrocyte layer and then a hard spin). Studies have shown that the single spin method packs erythrocytes to the bottom of the test tube to be discarded. A “hard spin” will also begin to separate leukocytes from platelets. The double spin allows for the platelets to be separated further from the plasma in the form of pellets for improved recovery (20, 21).

The make-up of an ideal PRP product (concentration of platelets, neutrophils, macrophages, total leukocytes, and erythrocytes) remains unknown. The purpose of this study was not to determine the makeup of an ideal PRP product, but rather to evaluate the cellular composition of the final product of the Stryker Regenkit PRP kit, specifically in regard to the platelet, RBC, and WBC concentrations. As future studies continue to gather evidence of what PRP product components are best for varying conditions, information garnered from the study reported here, can be used to choose which system would be best for particular syndromes.

## Materials and Methods

Ten adult healthy dogs were prospectively recruited for the study. Informed owner consent was obtained from all participating subjects and the study protocol was approved by the Animal Medical Center Institutional Animal Care and Use Committee. Dogs were considered for inclusion if no abnormalities were identified on physical examination by the primary investigator the same day of the study or abnormalities in CBC obtained within a month of the study. All subjects were > 15 kg in weight, neutered or intact with age range >1 yr <12 years. Normal pre-screening CBC was confirmed on the same day as study collection. Any subject who had significant alterations in CBC or that required sedation to obtain the necessary blood sample was excluded.

Once results of the pre-screening blood work returned with no abnormalities, adequate samples of blood (10 ml peripheral whole blood from the jugular vein) were obtained by one of two certified veterinary technicians. A sample of 2 ml of whole blood was submitted for a comprehensive CBC (Idexx® ^2^ test code 326) and 8 ml was used as per Stryker PRP Kit manufacturer guidelines as follows. The same investigator (BC) processed all the samples as described here per manufacturer instructions. The 8 ml of whole blood was placed in the RegenTHT tube and inverted gently 4–5 times to mix the anticoagulant additive with the blood. The RegenTHT tube was then inserted into the centrifuge with a measured counterbalance tube filled with an equal amount of water. The lid was closed and set for 1,500 Relative Centrifugal Force (3400 RPM) for 8 min. When the centrifugation was completed, the RegenTHT tube was removed and inverted 5 times to resuspend the platelets in the plasma supernatant. The supernatant fraction (final plasma product) was then collected with an 18 gauge needle on a 6 ml syringe. A final comprehensive CBC was then performed immediately on the final product from the Stryker kit. Comparisons in platelet, WBC, and RBC counts were made between the peripheral CBC (jugular blood collected at same time as the PRP sample) and the final Stryker plasma product for each dog.

### Statistical Methods

Baseline descriptive statistics are reported as mean and standard deviation. The distribution of residuals was assessed by visual inspection followed by Kolmogorov Smirnoff test. Between-groups and within group analyses were performed using independent and dependent model analysis of variance (ANOVA) where applicable as error residuals were normally distributed. All analyses were carried out using SAS 9.4 statistical software (Cary, NC) where *p* < 0.05 was deemed significant.

## Results

There was no statistically significant difference between concentrations of platelets for screening CBC on peripheral blood and the final plasma product (*p* = 0.3490). The mean platelet concentration on the peripheral blood samples was 258.8 K/uL while the mean concentration of platelets in the final product was 179.9 K/uL (Table 1). Furthermore, the results showed that not all final plasma products had higher platelet concentrations compared to the screening CBC for the same subject. Seven of the ten samples had lower concentrations of platelets in the final product, while only three had increased platelet concentrations in the final plasma product (Table 2).

**Table 1:** Mean and standard deviation cell counts in CBC of peripheral blood compared with CBC of final processed PRP product in 10 healthy canines using Stryker Regenkit PRP Kit.

	**Peripheral blood CBC**	**Final PRP product CBC**	***p*-value**
Platelets (K/uL)	258.8 ± 73.2	179.9 ± 249.0	0.349
Lymphocytes (/uL)	2765.7 ± 1141.8	2575 ± 1990.8	0.7957
Monocytes (/uL)	372.1 ± 157.7	279.9 ± 367.9	0.4758
Neutrophils (/uL)	5150.3 ± 1593.2	2303.9 ± 1335.0	0.0004
Red Blood Cells (M/uL)	7.2 ± 0.4	0.05 ± 0.04	<0.0001
White Blood Cells (K/uL)	8.6 ± 1.9	5.7 ± 3.5	0.0318

**Table 2:** Platelet counts in CBC of peripheral blood compared with platelet counts in final processed PRP product in healthy canines using Stryker Regenkit PRP Kit.

**Patient**	**Peripheral blood**	**Processed PRP**
	**Platelet count (K/uL)**	**Platelet count (K/uL)**
1	226	234
2	320	118
3	404	854
4	254	103
5	215	221
6	130	88
7	256	12
8	294	122
9	277	15
10	212	32

There were significantly less white blood cells found in the processed plasma product compared to screening CBC peripheral blood sample (*p* = 0.0318; Table 1). There were significantly less neutrophils found in the processed sample compared to the peripheral blood (*p* = 0.0004; Table 1). There was no statistically significant difference in monocytes or lymphocytes between the peripheral blood and processed plasma product (*p* = 0.4758 and 0.7957, respectively; Table 1).

The greatest difference between samples was found in red blood cell count. The mean concentration of red blood cells was significantly less in the final plasma product (0.045 M/uL) compared to peripheral blood (7.192 M/uL; *p* = < 0.0001; Table 1).

Despite there being two times as many males than females in the study population, the frequency of males (12) vs. females (22) was not found to be significantly different (*p* = 0.2059; Table 3).

**Table 3:** Subject demographics of adult healthy dogs used to test PRP product using Stryker Regenkit.

**Patient**	**Age (years)**	**Sex**	**Weight (kg)**
1	4.5	M	19.70
2	3	FS	23.10
3	9	FS	27.20
4	3	NM	19.80
5	2	NM	34.00
6	3	NM	24.70
7	10	NM	17.70
8	7	NM	35.50
9	4	NM	18.70
10	3	FS	17.60

The platelet:leukocyte and platelet:neutrophil ratios in the final plasma product were also evaluated between dogs (Table 4). The platelet:leukocyte ratio ranged from 2.54 to 108.1 with a mean of 46.4 and standard deviation of 55.5. The platelet:neutrophil ratio ranged from 0.005 to 0.27 with a mean of 0.091 and standard deviation of 0.092.

**Table 4:** Platelet:Leukocyte and Platelet:Neutrophil Ratios in the Final PRP Product of each dog and Mean ± Standard Deviation of ratios Using the Stryker Regenkit PRP Kit.

**Dog**	**Platelet:Leukocyte ratio**	**Platelet:Neutrophil ratio**
1	41.05	0.15
2	62.11	0.1
3	108.1	0.21
4	28.61	0.06
5	17.97	0.04
6	22	0.04
7	1.29	0.005
8	174.29	0.27
9	2.54	0.005
10	6.04	0.02
Mean ± SD	± 55.52	0.091 ± 0.092

## Discussion

In the present study, there was no significant difference in concentration of platelets when peripheral blood was compared to the final plasma product of the Stryker system. Previous literature suggests that a 3–5-fold increase in platelet concentration is desirable for an optimal PRP product (23). In the study performed here, the mean concentration of platelets following centrifugation was actually lower than the mean concentration of platelets in the screening CBC (Table 1). Only 3 of the 10 subjects demonstrated a final plasma product with a higher concentration of platelets when compared to the initial CBC on peripheral blood (prior to processing). Unlike the system studied here, Perego et al. (24) showed that the CPUNT 20 system consistently concentrates canine platelets. However, the Stryker RegenKit PRP system is not the only system of its kind that was shown not to concentrate platelets efficiently. In a study by Carr et al. (12) two of the five systems investigated did not see significant changes in PRP platelet concentrations compared to whole blood. Similarly, Franklin et al. (18) found one of five systems evaluated did not concentrate platelets. The product tested in this study may therefore not provide the suspected beneficial effects of healing, tissue regeneration, and analgesic effects brought about by the presence of platelets in a final PRP concentrate (10, 25).

The current study showed that the final plasma product of the Stryker system resulted in significantly less white blood cells compared to peripheral blood. This is similar to two of five systems in one study (12) and two of five in another (18). In a study carried out by Perego et al. (24) evaluating the CPUNT 20 system, the final product did not have a significantly different leukocyte count compared with whole blood. This system; however, has two settings which gives the user the option of allowing or disallowing leukocytes in the final product. From previous studies, we know that leukocytes lead to catabolism of tissue while also promoting an inflammatory response (10, 26). In cases with infected wounds, it may be beneficial to introduce leukocytes to the site of healing (4). Increases in matrix metalloproteinase-13 also promotes degradation of tendon and ligament (10, 26). Braun et al. (22) showed increased pain, cell death, and synoviocyte activation in PRP products what were leukocyte rich (22). Therefore, increased leukocyte counts would likely be less ideal in a final PRP product being used for tendon, ligament, bone, and/or osteoarthritis cases (4, 10, 14, 26).

There were less neutrophils following processing in the Stryker system compared to peripheral blood; however, there was no significant difference in concentrations of monocytes or lymphocytes. To draw a comparison between the system evaluated here to those from previous studies, three of five systems examined by Carr et al. (12) and the system evaluated by Perego et al. (24) also significantly reduced neutrophil concentrations. Only two of the five systems final products examined by Carr et al. (12) had no significant change of monocyte concentration compared to whole blood (two had significantly decreased concentrations and one had an increased concentration) and all had significantly different concentrations of lymphocytes compared to whole blood (two had significantly decreased concentrations and three had an increased concentration). The CPUNT 20 system's final PRP product had an increased concentration of both monocytes and lymphocytes when compared to whole blood (24). This shows the vast variability of numbers and types of leukocytes present across the various final products produced by the different machines. It is suspected that neutrophils would be beneficial for cases involving infectious processes; however, they can also cause undesirable tissue destruction. Monocytes, on the other hand, divide into macrophages and may be desirable in a PRP product meant for musculoskeletal repair due to their ability to clear harmful debris from injured tissue and other anabolic effects (10).

The study reported here found significantly less red blood cells in the Stryker plasma product when compared to peripheral blood. This seems to be fairly consistent across many PRP systems as all systems evaluated by Carr et al. (12) and Perego et al. (24) also significantly reduced erythrocyte concentrations in the final product when compared to whole blood. Franklin et al. (18) did not report erythrocyte count, rather hematocrit and therefore was not compared to the results of this study. It remains unknown if an ideal platelet rich plasma product should be devoid of red blood cells. Previous studies report that RBCs may cause direct cartilage damage, stimulate synoviocytes to release catabolic mediators, and may be cytotoxic due to free radical induced apoptosis (10, 22). Despite possible benefits of their ability to bring oxygen to tissues and promote vasodilation, it is likely that they are undesirable for most cases of musculoskeletal injury.

The results of this study indicate that the final product of the Stryker RegenPRP Kit did not produce what can be defined as a PRP product from canine blood. As it was unable to consistently concentrate platelets, it does not fall into one of the four general PRP categories (P-PRP, L-PRP, P-PRF, and L-PRF). We suspect that this is due to the processing with a single 8-min “soft” spin session at 1,500 g. Had a harder spin been performed, better separation of platelets from leukocytes may have been appreciated. Had second spin been performed, better and more efficient separation of platelets may have been possible. However, additional studies would need to be performed to substantiate these assumptions. Using the classification system discussed in the introduction, the final Stryker Regenkit plasma product would be considered closest to the Leukocyte Poor PRP (LP-PRP) as all final plasma products contained lower numbers of neutrophils compared to the peripheral blood cell count following processing. This classification of product is similar to the final product produced by two of the five systems evaluated by Frankin et al. (27).

In the presented study, no consistency was found between dogs for either platelet:leukocyte ratio or platelet:neutrophil ratio. Franklin et al. (18) discusses consistency of platelet:leukocyte ratios across 5 different PRP machines; however, no studies could be found by the author that evaluates these ratios between canine samples using the same machine.

It is worth noting that no previous validation studies for canines or any other species using the Stryker Regekit PRP Kit could be found on literature search. Requests to the manufacturers requesting the data and studies were not answered. As no previous literature could be found on the Stryker Regenkit PRP system in any species, the authors were unable to compare the findings of this study with those of others performed. A major limitation of this study is the small number of dogs used. This may lead to type II errors in our data. Further studies with increased number of subjects would be ideal.

As ideal PRP product compositions are learned for differing conditions, the authors feel it is important that any company manufacturing such a system perform, and make available, a 3rd party prospective analysis of their biologics/devices prior to advertisement and sale. It is also recommended that clinicians perform and record pre- and post-spin analysis on all PRP products that are processed to ensure the most appropriate preparation is being used and can be tracked over time.

## Data Availability Statement

The raw data supporting the conclusions of this article will be made available by the authors, without undue reservation.

## Ethics Statement

The animal study was reviewed and approved by Animal Medical Center Institutional Animal Care and Use Committee.

## Author Contributions

BC, LA, and SC contributed to conception and design of the study. BC organized the database. BC and LA wrote the manuscript. KL provided statistical analysis. All authors contributed to manuscript revision, read, and approved the submitted version.

## Conflict of Interest

SC is a paid consultant of Companion Animal Health, who produces a PRP kit and could be construed as a competitor to Stryker. KL is employed by the company Lamb Statistical Consulting LLC. The remaining authors declare that the research was conducted in the absence of any commercial or financial relationships that could be construed as a potential conflict of interest.
